# Factors associated with mental health symptoms among UK autistic children and young people and their parents during the COVID-19 pandemic

**DOI:** 10.1177/13623613231153694

**Published:** 2023-02-27

**Authors:** Melanie Palmer, Susie Chandler, Virginia Carter Leno, Farah Mgaieth, Isabel Yorke, Matthew Hollocks, Andrew Pickles, Vicky Slonims, Stephen Scott, Tony Charman, Emily Simonoff

**Affiliations:** 1 King’s College London, UK; 2 Guy’s and St Thomas’ NHS Foundation Trust, UK; 3 South London and Maudsley NHS Foundation Trust, UK

**Keywords:** autism, children and young people, COVID-19 pandemic, mental health, parents

## Abstract

The current study explored the role of pre-existing and pandemic-time child, family or environmental factors in the presentation of mental health symptoms of autistic youth and their parents during the pandemic. Participants were parents/carers of autistic children (Autism Spectrum Treatment and Resilience Cohort, *N* = 67, *M*_age_ = 9 years) and adolescents (QUEST cohort, *N* = 112, *M*_age_ = 17 years). Parents completed an online survey that asked about child and parental mental health, infection experience, and changes to education arrangements, family life, housing and finances during the pandemic. Pre-existing measures of mental health, autism and adaptive functioning were also utilised. More engagement and enjoyment in education provision and going outside was associated with better child and parental mental health. In multivariate multiple linear regression models, more pre-existing attention deficit hyperactivity disorder symptoms were associated with more behavioural/attention deficit hyperactivity disorder symptoms during the pandemic in the pre-adolescent cohort, and with greater emotional symptoms in the adolescent cohort. More pre-existing parental mental health problems were associated with more parental mental health symptoms during the pandemic in both cohorts. Knowledge of pre-existing mental health and pandemic-related stressors may help care planning. Encouraging engagement and enjoyment in education and promoting physical exercise are key intervention targets. Ensuring access to attention deficit hyperactivity disorder medication and support is important, especially if this is managed jointly across school and home.

**Lay abstract:**

**What is already known about the topic:** The COVID-19 pandemic and the associated restrictions impacted all of society. There is emerging evidence showing a range of impacts on autistic children and young people and their families. Further research that looks at how individuals coped during the pandemic while considering how they were doing before the pandemic is needed.

**What this paper adds:** This article explores whether how well autistic youth were doing before the pandemic influenced how they coped during the pandemic. It also looked at how well their parents were doing during the pandemic and whether any pre-pandemic factors influenced how they coped. Samples of both primary-school-aged autistic children and autistic teenagers and their parents were surveyed to answer these questions. More engagement and enjoyment in education provision during the pandemic and getting outside more were linked with better child and parental mental health during the pandemic. More attention deficit hyperactivity disorder before the pandemic was linked with more attention deficit hyperactivity disorder and behavioural problems during the pandemic in primary-school-aged autistic children, and more emotional problems during the pandemic in autistic teenagers. Parents with more mental health problems during the pandemic had more mental health problems before the pandemic.

**Implications for practice, research or policy:** Encouraging engagement and enjoyment in education and promoting physical exercise are key intervention targets. Ensuring access to attention deficit hyperactivity disorder medication and support is important, especially if this is managed jointly across school and home.

## Introduction

### Impact of the COVID-19 pandemic on children and young people

The COVID-19 pandemic had a widespread impact on society worldwide. Three key vulnerable groups were: children and young people (CYP) and families affected by school closures; those with pre-existing mental health symptoms, broadly known as a latent trait of psychopathology (Caspi et al., 2014) (e.g. emotional symptoms such as those associated with depression and anxiety, and behavioural symptoms such as those associated with disruptive behaviour); and individuals with neurodevelopmental disorders or learning difficulties (Holmes et al., 2020; Novins et al., 2021; Vasa et al., 2021). A systematic review of the literature investigating mental health problems in CYP during restrictions found that between 31% and 42% had emotional symptoms, behavioural or attention deficit hyperactivity disorder (ADHD) symptoms, and in 79% of CYP their behaviour/psychological state was negatively affected. Findings from the Co-SPACE study, a large-scale general population longitudinal study tracking the trajectories of mental health symptoms of CYP aged 4–16 years in the United Kingdom, reported poorer child mental health during the early phases of the pandemic when schools were closed and strict social restrictions applied (Raw et al., 2021; Waite et al., 2021). This was particularly true for pre-adolescents (4–10-year-olds) where probable clinically elevated levels of emotional symptoms, behavioural symptoms and ADHD increased by 10%, 35% and 20%, respectively, and was greater in girls than boys. Among adolescents, there were no significant changes in the proportion meeting clinically elevated levels for these symptom domains. Findings from England’s Mental Health of Children and Young People (MHCYP) survey, a large nationally representative cohort, also indicated an increase in the incidence of mental health symptoms from 10.8% in 2017 to 16.0% during the pandemic among 5- to 16-year-olds (Newlove-Delgado et al., 2021). This increase held across age, gender and ethnic group.

Autistic CYP were particularly at risk for negative impacts of the pandemic for several reasons (Aishworiya & Kang, 2020). First, autistic CYP, of which about 35%–50% have an intellectual disability (ID) (Charman et al., 2011; Christensen & Zubler, 2020), already have much higher rates of mental health problems when compared to neurotypical children (Lai et al., 2019; Simonoff et al., 2008). Before the pandemic, as many as 80%–90% of autistic CYP had a co-occurring psychiatric diagnosis, the most common being an anxiety disorder, ADHD or a disruptive behaviour disorder. Second, 10%–15% display behaviour that means they cannot attend mainstream schools and participate in family and community life in the usual ways (Emerson et al., 2001). Third, intolerance to uncertainty has been suggested as a causal model for why emotional symptoms like anxiety are heightened among autistic individuals (Boulter et al., 2014; Jenkinson et al., 2020). Additional cognitive deficits such as impaired information processing, emotional insight and difficulties with goal-directed behaviour, may further reduce autistic CYP’s adaptive strategies during times of change/uncertainty and in turn lead to a trauma-type response (Mazefsky et al., 2013; Wood & Gadow, 2010), manifested by mental health symptoms. During the pandemic and associated restrictions, uncertainty about all aspects of daily life was commonplace and the requirement to adapt routines, especially with school closures, could have led to increases in mental health symptoms and place strain on family relationships (Buchnat & Wojciechowska, 2020; Patel et al., 2020). Finally, autistic CYP may have been disproportionately disadvantaged by the impacts of the lockdowns due to an increased reliance on community and school-based services for treatment (i.e. therapies) and enrichment (i.e. extra-curricular activities) (Aishworiya & Kang, 2020; Ameis et al., 2020) and social care.

However, findings from studies exploring the mental health of CYP with neurodevelopmental disorders during the pandemic present a varied picture. For example, the Co-SPACE study showed children with special educational needs (SENs) or neurodevelopmental disorders to have markedly elevated levels of emotional symptoms, behavioural symptoms and ADHD during the pandemic, compared to those without SEN or neurodevelopmental problems (Waite et al., 2021). Since the study did not include a measure of pre-pandemic symptoms, it is not clear whether this difference is related to the pandemic or simply a reflection of higher pre-existing symptoms in the participating volunteer sample. Looking at change over time, Co-SPACE found a slight *decrease* in parent-reported emotional, behavioural and ADHD symptoms in SEN CYP over the first UK lockdown; whereas a slight *increase* in these symptoms was observed among CYP without SEN. In Toseeb and Asbury (2022), autistic young people reported more emotional symptoms, like anxiety and depression, compared to children with other disabilities, and that anxiety symptoms remained stable even after schools reopened. Another study, comparing mental health symptoms in CYP with intellectual disability during the pandemic found similar levels of symptoms before the pandemic (Bailey et al., 2021). Finally, and of particular relevance to the current study, are the findings from a US clinical sample of autistic children showing a worsening in mental health and/or development of new mental health symptoms among 59% of their sample during the pandemic (Vasa et al., 2021). Risks for worsening mental health included the child’s understanding of COVID-19, low family income and greater pandemic-time parental distress (Vasa et al., 2021).

### Impact of the COVID-19 pandemic on parents/carers

The findings from studies exploring the mental health of parents during the pandemic also are mixed. A systematic review of literature reporting mental health symptoms in parents/carers of CYP reported that 52% had anxiety and 27% had depression during the pandemic (Panda et al., 2021). In Co-SPACE, a significant rise in family-related stress early in the pandemic was found (Shum, Skripkauskaite, Pearcey, Raw, et al., 2021), with parental anxiety, stress and depression surpassing levels seen early in the pandemic when restrictions were re-introduced in November 2020 (Shum, Skripkauskaite, Pearcey, Waite et al., 2021). Parents of autistic CYP were particularly vulnerable as they already had higher levels of parenting stress than parents of children with other developmental disabilities and those without neurodevelopmental difficulties (Estes et al., 2013), and there is an association between greater parenting stress and child mental health problems (Yorke et al., 2018). A systematic review of the literature on the mental health of parents of autistic CYP demonstrated increases in parental anxiety and stress and difficulties coping (Yılmaz et al., 2021). One study found that when compared to parents of non-autistic children, parents of autistic CYP reported higher levels of overall psychological distress and more feelings of panic when thinking about the pandemic (Kalb et al., 2021), but another found that the mental health symptoms of parents of autistic young people did not differ from those with other disabilities (Toseeb & Asbury, 2022). Among UK parents of SEN children, a study found that most parents reported changes in mental health symptoms related to social restrictions, many felt overwhelmed and only a minority reported there was little impact on mental health in their family (Asbury et al., 2021). When comparing pre-pandemic parental psychological distress to distress during the pandemic, Bailey et al. (2021) found that UK parents with children with an intellectual disability reported similar levels of parental psychological distress at both timepoints. However, other research has reported much greater levels of anxiety and depression during the pandemic among carers of children with an intellectual disability when compared to parents of children without intellectual disability (Willner et al., 2020). Another study comparing carers of autistic and non-autistic individuals did not find significant differences in mental health symptoms during the pandemic between these groups (Fusar-Poli et al., 2022). Risk factors for more parental mental health symptoms during the pandemic have included lower pre-pandemic family income and more pandemic-related stressors (e.g. loss of occupation) (Lengua et al., 2022).

### Research objectives

COVID-19-related research conducted with CYP with neurodevelopmental disorders has mainly surveyed or tracked CYP since the pandemic began. While this provides essential information about the current mental health of this population and how it has changed over the course of the pandemic, the lack of systematically selected samples means that there is limited understanding of participant representativeness, and the lack of pre-pandemic measures of mental health symptoms does not allow for comparisons to symptoms during the pandemic or identification of risk and protective factors. Further research examining who is most at risk of negative impacts of the pandemic based on pre-existing factors and lockdown circumstances is needed. Such information is essential to understand whether there are sub-groups who may be more adversely affected and may in turn be used to guide future care planning and pandemic response.

The current study aims to address the following research questions:

*Research Question 1.* How did the pandemic impact the lives of families of autistic children?
*Research Question 2*. Were the mental health symptoms of autistic CYP during the pandemic associated with pre-existing mental health symptoms, over and above autism characteristics, adaptive functioning and other contextual factors related to the pandemic?
*Research Question 3.* Were the mental health symptoms of parents/carers of autistic CYP during the pandemic associated with their own/their child’s pre-existing mental health symptoms, over and above their child’s autism characteristics, adaptive functioning and other contextual factors related to the pandemic?

We included two samples of well-characterised pre-adolescent and adolescent CYP to examine impact across the developmental period and allow for internal replication of results. Both samples were originally recruited from a similar geographical area and tracked over time as part of the wider Improving Autism Mental Health (IAMHealth) programme and hence policies around education and social restrictions will have been similar for both cohorts. Comparing the cohorts in this way enabled us to look at the commonalities and differences that apply to autistic CYP at two important developmental stages during the pandemic.

We hypothesised that greater autism characteristics, pre-existing mental health and behavioural symptoms and environmental stressors would be associated with increased mental health symptoms during the pandemic among both pre-adolescent and adolescent CYP. For parents, we hypothesised that those with CYP with more pre-existing mental health symptoms, those who had more pre-existing parental mental health symptoms and those with pandemic-related environmental stressors would report more parental mental health symptoms during the pandemic.

## Method

### Sample and procedure

**Figure 1.**
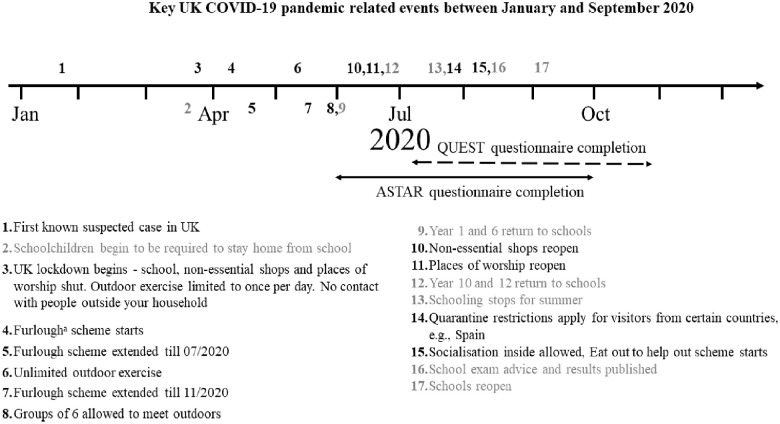
Key COVID-19 pandemic-related events in the United Kingdom and data collection periods. *Note.* ^a^Non-essential businesses unable to operate due to restrictions. Staff could be put on temporary leave and have wages covered by the government. Events in black impact all of society. Events in grey relate to schooling.

The current study consists of two samples of autistic CYP drawn from the South London area. Separate public involvement panels of autistic adults and parents of autistic children advised on the research conducted with both cohorts. This involvement consisted of seeking guidance on all aspects of the research, from objectives to design and interpretation of findings via regular group meetings and electronic feedback. Data collection for the current study was conducted early in the pandemic. Pre-existing measures were taken pre-pandemic. Figure 1 displays the data collection timeline and COVID-19-related events. Further information on the two samples is provided in Table 1, and the recruitment of the samples in the Supplementary Materials.

**Table 1.** ASTAR and QUEST sample characteristics.

	ASTAR (*N* = 67)	QUEST (*N* = 112)
% of contacted sample participating	82% participated	53% participated
Mean age in years (*SD*), range	8.8 (1.49), 6.0–11.8	17.9 (1.15), 15.9–20.1
Sex: *N* (%) male	56 (84%) male	89 (79%) male
Ethnicity: *N* (%) White	34 (52%) White^a^	59 (54%) White
School type: Mainstream: *n* (%) and special education	38 (58%) mainstream, 28 (42%) special education^a^	48 (51%) mainstream, 46 (49%) special education
Mean ABAS GAC (*SD*), range	61.2 (12.6), 45–100^a^	60.6 (29.2), 23–105
Mean SCQ (*SD*), range	23.8 (6.87), 9–34^a^	17.6 (6.68), 3–35^c^
Mean SCQ SCI (*SD*), range	15.3 (5.5), 4–25^a^	10.9 (5.1), 1–21^c^
Mean SCQ RRB (*SD*), range	7.4 (2.5), 1–12^a^	5.7 (3.1), 0–12^c^
Mean ADOS CSS (*SD*), range	7.5 (1.82), 1–10^a^	6.5 (2.72), 1–10^d^
Mean ADOS SSA CSS (*SD*), range	7.9 (1.8), 1–10^a^	7.2 (2.4), 1–10^d^
Mean ADOS RRB CSS (*SD*), range	6.7 (2.4), 1–10^a^	4.7 (3.2), 1–10^d^
Parent/carer informant		
Mother: *n* (%)	62 (92.5%)	107 (95.5%)
Father: *n* (%)	4 (6.0%)	4 (3.6%)
Grandmother: *n* (%)	1 (1.5%)	1 (0.9%)
Parental Education:		
At least one parent with A-levels or above: *n* (%)	58 (87.8%)^a^	64 (60%)^b^
Parental Employment prior to pandemic		
At least one parent in employment: *n* (%)	54 (81.8%)^a^	79 (79%)^c^

ASTAR: Autism Spectrum Treatment and Resilience; ABAS GAC: Adaptive Behaviour Assessment System General Adaptive Composite; CSS: Calibrated Severity Score; RRB: Restricted and Repetitive Behaviours; SCI: Social Communication and Interaction; SCQ: Social Communication Questionnaire.

a Available on *n* = 66.

b Available on *n* = 107.

c Available on *n* = 100.

d Available on *n* = 44.

#### ASTAR cohort (enrolled in a feasibility pilot randomised controlled trial)

The Autism Spectrum Treatment and Resilience (ASTAR) cohort consisted of 85 parents of young autistic children who consented to participate in a feasibility study or pilot randomised controlled trial of novel group-based parent-mediated interventions (ISRCTN91411078), as part of IAMHealth (Charman et al., 2021). All parents from the ASTAR cohort who had consented to future research contact and had a valid email address (*N* = 82/85, 96%) were invited to participate in the current study via email (June–September 2020; see Figure 1). The invite included a link to a secure online platform giving families access to the study information sheet, consent form and the COVID survey (described below). Researchers followedup non-responding families via phone. The current study was reviewed and given ethical approval from the Psychiatry, Nursing and Midwifery Research Ethics Subcommittee at King’s College London (REMAS ref: 19146, ethical clearance ref: HR-19/20-19146). Informed consent was collected from all participating families.

The sample participating in the current study consisted of 67 parents (92.5% mothers) of autistic children. Most children were male (83.6%) and were on average 8 years 10 months (range 6:0–11:10) during the pandemic-time data collection. Thirty-five (52.2%) children were minimally verbal and 32 (47.8%) were verbal, as verbal ability was stratified for in the original study design. The children were split across specialist (42.4%) and mainstream (57.6%) education provision when originally assessed.

#### QUEST cohort

All parents/carers from the QUEST cohort (Salazar et al., 2015) who had consented to future research contact and had a valid email address (*N* = 211) were invited to participate in the current study. In previous waves of data collection for the QUEST cohort, children were split into an ‘intensively studied’ (hereafter intensive) and ‘extensively studied’ group (hereafter extensive). The intensive group completed in-depth assessments (including direct observations of the CYP) whereas the extensive group completed shorter online parent-report questionnaires only. For the current study, families were approached and data was collected in the same manner as described above for the ASTAR cohort. All parents/carers completed the same measures, with data collection taking place between July and October 2020 (see Figure 1). Ethical approval was granted by Camden and King’s Cross Research Ethics Committee for the current study (ref: 20/LO/0625). Informed consent was collected from all participating families.

From the 211 contacted, 112 (53%) parents/carers (95.5% mothers) participated. 79% of their CYP were male; 37% had an IQ <70 when assessed pre-pandemic; and 49% were in specialist education at the last point of data collection approximately 18 months prior to the pandemic.

## Measures

### COVID survey

The European Child & Adolescent Psychopharmacology Network (ECNP) Child and Adolescent Mental Health Services (CAMHS) COVID survey was designed to tap into a broad range of mental health symptoms experienced during the pandemic and contextual factors particularly pertinent to periods of social restrictions. Questions from the Coronavirus Health and Impact Survey mental health section (Nikolaidis et al., 2020) were used to inform the questions about mental health, which focussed on symptoms in the past two weeks. The questionnaire also asked about COVID-19 infection in the child, nuclear and wider family, and child and parental worry about becoming infected. Contextual factors included information on parental stress, parent-child relationships, parental partner relationships, employment and financial stress, adequacy of the home environment and educational provision.

Factor analysis of this measure using a larger dataset collected from CYP in South London and Maudsley (SLaM) CAMHS (manuscript in preparation) led us to adopt a two-factor solution for both CYP and parent mental health symptoms, which performed well (see Supplementary Materials for loadings for the current sample). The questions on the child’s emotional and behavioural symptoms formed two factors: (1) emotional symptoms (six items measuring general worry, anxiety, sadness, loneliness, enjoyment of usual activities and tiredness each on a 5-point scale) and (2) behavioural/ADHD symptoms (four items measuring irritability/anger, aggressiveness, restlessness and concentration on a 5-point scale). The questions on parental emotional and behavioural symptoms formed two factors: (1) emotional symptoms (measured using the same six items) and (2) irritable behaviour (three items measuring irritability/anger, restlessness and concentration on a 5-point scale).

A range of other measures collected in the ASTAR and QUEST cohorts at earlier timepoints were used to explore the relationship between pre-existing vulnerabilities and the additional stressors of COVID-19 on CYP and parental mental health. These are listed in Supplementary Table 3 and assessed the constructs as described below.

### Autism characteristics

The Social Communication Questionnaire (SCQ, Rutter et al., 2003) was completed by parents in the ASTAR (Lifetime version) and QUEST (Current version) cohorts. SCQ total scores were generated, as well as domain scores for social communication and interaction impairment (SCI) and for restricted and repetitive behaviour (RRB) as recommended by Evans et al. (2019), with higher scores indicating more characteristics. The Autism Diagnostic Observation Schedule–2 (ADOS–2, Lord et al., 2012) was used with CYP in both ASTAR and QUEST (intensives only), as a direct observation of autism characteristics. Calibrated severity scores (CSS), which take into account age and language level (Hus et al., 2014), were calculated for the social affect (SA) and RRB domains.

### Adaptive functioning

The Adaptive Behaviour Assessment System (ABAS) was used as a parent-report measure of adaptive functioning in ASTAR (ABAS-III, Harrison & Oakland, 2015) and QUEST (ABAS-II, Harrison & Oakland, 2003). The ABAS asks parents for information about their child’s skills across a range of domains, and these domain scores are then used to generate a general adaptive composite (GAC) score with higher scores reflecting higher ability levels. In the QUEST cohort, the intensive sample completed the full ABAS, while the extensive sample completed items from the communication domain only. Scores for the other eight skill domains were calculated using multiple imputation, and these were used along with the communication score to generate the GAC score for the QUEST extensives (see Supplementary Material for further information on the multiple imputation technique used to generate ABAS GAC scores for the extensives).

### Pre-existing CYP mental health problems

In the ASTAR cohort, pre-existing parent-reported CYP mental health and behavioural symptoms were measured using the: (1) Preschool Anxiety Scale–Revised (PASR, Edwards et al., 2010), a 28-item measure which taps into specific fears, and generalised, social and separation anxiety; (2) Aberrant Behaviour Checklist–Hyperactivity subscale (ABC, Aman, 2013), 16 items measuring hyperactivity and impulsivity; and (3) Home Situations Questionnaire–Autism Spectrum Disorders (HSQ-ASD, Chowdhury et al., 2015), a 24-item autism-specific measure of the severity of child non-compliance in everyday situations, with higher scores on all measures indicating more emotional symptoms, ADHD and behavioural symptoms, respectively.

In the QUEST cohort, pre-existing CYP mental health and behavioural symptoms were measured using the Strengths and Difficulties Questionnaire (SDQ, Goodman et al., 2000). The SDQ comprises 25 items from which a total score and five subscales are derived, with higher scores indicating more problems. In the current study, the Emotional Symptoms, Hyperactivity-Inattention and Conduct Problems subscales were used as measures of prior emotional symptoms, ADHD and behaviour symptoms, respectively.

### Pre-existing parental distress

The Autism Parenting Stress Index (APSI, Silva & Schalock, 2012) was used in ASTAR to measure pre-existing parenting stress. The APSI consists of 13 items assessing family stress related to autism characteristics and co-morbid problems, with higher scores indicating greater parenting stress. The Kessler Psychological Distress Scale (K-10, Kessler et al., 2002) was used in QUEST to measure pre-existing parental psychological distress. The K-10 includes 10 questions about cognitive, behavioural, emotional and psychophysiological symptoms, with higher scores indicating a higher level of psychological distress.

### Statistical analysis

Data analysis was conducted in Stata MP 16/17 (StataCorp, 2015). All variables were assessed for normality. Multivariate multiple linear regression was used to test the associations between the child, family and environmental factors and mental health symptoms during the pandemic. As shared constructs were measured in both cohorts, we followed a similar analytic approach in both samples but conducted the analysis separately for ASTAR and QUEST.

The bivariate associations between the independent and dependent variables were first examined (See Supplementary Table 4). Variables that were significant at *p* < .05 in either cohort were entered into a multivariate multiple regression model, using structural equation modelling with full information maximum likelihood. This analysis was chosen to generate standardised coefficients for comparison across the samples and deal with missing data. In the models examining CYP symptoms, the dependent variables were CYP emotional symptoms or CYP behavioural/ADHD symptoms (as measured by the COVID survey). Independent variables included: CYP age, level of adaptive functioning, autism characteristics, pre-existing CYP mental health problems, pre-existing parental distress and the family’s current financial situation, home accommodation, access to garden and CYP physical attendance at school/college. In addition to these, level of adaptive functioning and autism characteristics were retained as co-variates to account for CYP ability levels and autism characteristics, even if they weren’t significant predictors at the bivariate level.

In the models examining parent symptoms, again any variables with a significant bi-variate association with any pandemic-time outcome at *p* < 0.05 were entered into a multivariate multiple regression. The dependent variables were: parent emotional symptoms or parent irritable behaviour (as measured by the COVID survey). The independent variables were the same as those specified in the CYP outcome models above.

## Results

### Attrition

For both cohorts, there were no significant differences between participants and non-participants with regard to child and parent characteristics, with exception of parental unemployment being more common among QUEST non-participants versus participants (see Supplementary Material for further information).

### Impact of the pandemic on family life

Exposure to COVID-19 was low in both samples in the 2 weeks prior to survey completion (see Table 2 for rates of infection). Access to education was restricted for the majority of CYP during the early phases of the pandemic: only 13% of CYP in ASTAR and QUEST experienced no change. Just half (53%) of the 6- to 11-year-olds in ASTAR and only a quarter (25%) of the 15- to 20-year-olds in QUEST were attending their school/college/apprenticeship placement in person, either on a part-time or full-time basis (see Table 2); but attendance was not associated with the level of either CYP or parent mental health symptoms during the pandemic in either sample (see Supplementary Table 4). However, less engagement and enjoyment in education was associated with more CYP emotional symptoms in ASTAR, more behavioural/ADHD symptoms in the CYP in both samples and more parental emotional symptoms in QUEST (see Table 3). A large proportion of CYP in ASTAR and QUEST were going outside their home less than once a week (28% and 21% respectively), with less time outside associated with greater mental health symptoms in adolescents and their parents. Parents’ working patterns also changed (see Table 2), for example, a substantial proportion of parents were furloughed (12% in ASTAR, 20% in QUEST; see Figure 1 for the definition of furlough).

**Table 2.** COVID-19 impact data from ASTAR and QUEST.

		ASTAR (*N* = 67)	QUEST (*N* = 112)
Exposure to COVID-19	Positive test in immediate family: *N* (%)	4 (6.0%)	5 (4.5%)
	CYP suspected, not tested: *N* (%)	2 (3.0%)	1 (0.9%)
	CYP suspected with a positive test: *N* (%)	0 (0.0%)	2 (1.8%)
	CYP suspected with a negative test: *N* (%)	1 (1.5%)	8 (7.1%)
	Death in the wider family as a result of COVID-19: *N* (%)	1 (1.5%)	2 (1.8%)
Parental working situation during pandemic	At least one parent furloughed due to COVID-19: *N* (%)	11 (16.4%)	22 (19.6%)
	At least one parent dismissed due to COVID-19: *N* (%)	4 (6.0%)	9 (8.0%)
	At least one parent working more hours than usual: *N* (%)	9 (13.4%)	7 (6.3%)
	At least one parent working less hours than usual: *N* (%)	7 (10.5%)	17 (15.2%)
	Level of financial concern: mean (*SD*), range	2.16 (0.95), 1–5	2.59 (0.80), 1–5
CYP education	CYP in person attendance at school/college at least on some days: *N* (%)	34 (50.8%)	13 (24.5%)^a^
	CYP enjoyment in education: mean score (*SD*), range	3.28 (1.28), 1–5	2.64 (0.97), 1 – 5
	CYP engagement in education: mean score (*SD*), range	2.67 (1.35), 1–5	2.49 (1.07), 1 – 5
Home environment	Have access to personal garden/outside space: *N* (%)	58 (86.6%)	83 (74.8%)^b^
	Cramped home environment: mean score (*SD*), range	1.85 (0.97), 1–4	2.21 (0.79), 1–4
CYP getting outside home for approved activities^c^	CYP getting outside: mean score (*SD*), range	2.21 (0.99), 1–4	2.15 (0.78), 1–4
CYP mental health	CYP emotional symptoms total	15.97 (4.75), 8–25	15.37 (4.25), 7–29
	CYP behaviour and ADHD symptoms total	12.06 (3.42), 4–18	9.84 (3.35), 4–20
Parental mental health	Parent emotional symptoms total	19.42 (4.98), 8–30	16.90 (3.56), 7–30
	Parent irritable behaviour total	7.93 (2.54), 3–15	6.67 (2.14), 3–15

ASTAR: Autism Spectrum Treatment and Resilience; CYP: children and young people.

*Note.* See Supplementary Materials for more details of the measures used.

a Based on the 53 QUEST families who completed survey prior to schools/colleges re-opening in September 2020.

b Available on *n* = 111

c Approved activities during the early phases of the pandemic included physical exercise, grocery shopping, attending medical appointments, or to go to school.

**Table 3.** Bi-variate relationships between COVID-19 educational impact questions and CYP and parent mental health symptoms for ASTAR and QUEST.

	ASTAR				QUEST			
	CYP Emotional Symptoms	CYP Behavioural/ADHD Symptoms	Parent Emotional Symptoms	Parent Irritable Behaviour	CYP Emotional Symptoms	CYP Behavioural/ADHD Symptoms	Parent Emotional Symptoms	Parent Irritable Behaviour
	Std Coeff.	Std Coeff.	Std Coeff.	Std Coeff.	Std Coeff.	Std Coeff.	Std Coeff.	Std Coeff.
CYP COVID impacts								
Enjoyment in education	−0.28*	−0.14	−0.04	0.08	−0.18	−0.28**	−0.26**	−0.11
Engagement in education	−0.24*	−0.33**	−0.06	−0.18	−0.08	−0.32***	−0.39***	−0.17
Getting outside	−0.11	−0.09	−0.34	0.01	−0.18	−0.24**	−0.37***	−0.28**

* *p* < 0.05. **p < 0.01. ***p < 0.001.

### Factors associated with CYP mental health symptoms during the pandemic

**Figure 2.**
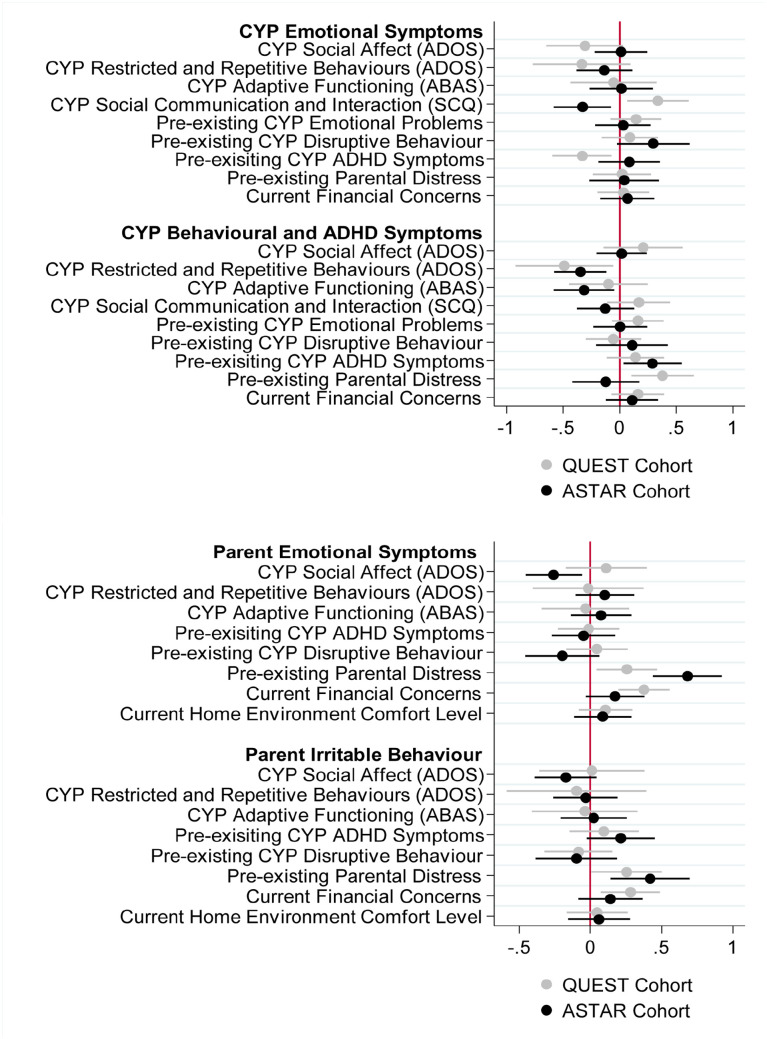
Forest plots showing the standardised coefficients for the multivariate multiple regression models predicting CYP and parent mental health symptoms. The *x* axis is the standardised coefficients and *y* axis displays each of the factors examined.

Figure 2 shows the standardised coefficients for factors associated with CYP mental health symptoms during the pandemic (see Supplementary Table 5 for details of the standardised coefficients and *p* values). Consistent across cohorts, lower pre-existing observed restricted and repetitive behaviour was associated with more behavioural/ADHD symptoms during the pandemic. However, lower pre-existing parent-reported social communication impairment scores were associated with more CYP emotional symptoms in ASTAR during the pandemic consistent with the observed restricted and repetitive behaviour association; whereas the opposite pattern was found in QUEST with higher parent-reported social communication impairment scores being associated with CYP emotional symptoms. Furthermore, in ASTAR, lower pre-pandemic adaptive functioning and increased pre-existing CYP ADHD behaviours were also associated with greater CYP behavioural/ADHD symptoms during the pandemic. In QUEST, higher levels of pre-existing parental distress were additionally associated with more pandemic-time behavioural/ADHD symptoms in the CYP. Pre-existing CYP emotional symptoms was not associated with pandemic-time emotional symptoms in neither sample, although more pre-existing ADHD was associated with greater emotional symptoms during the pandemic in QUEST.

### Factors associated with parental mental health symptoms during the pandemic

Figure 2 also shows the standardised coefficients of factors associated with parental mental health symptoms (see Supplementary Table 6 for details). Consistent across both ASTAR and QUEST, greater pre-existing parental distress was associated with increased parent emotional symptoms and increased parent irritable behaviour during the pandemic. For QUEST only, increased current financial concerns were also associated with increased parent emotional symptoms and irritable behaviour during the pandemic. In ASTAR, lower pre-existing child-observed SA was associated with greater pandemic-time parental emotional symptoms. However, no child factors were associated with parental mental health symptoms during the pandemic in QUEST.

## Discussion

The current study explored whether mental health symptoms in autistic CYP and their parents during the pandemic were associated with pre-existing mental health symptoms, over and above pre-existing measures of autism characteristics and adaptive functioning, and other contextual factors related to the pandemic. We utilised previously studied cohorts of both pre-adolescent and adolescent autistic CYP to examine the impact of pre-existing and current vulnerabilities across childhood.

### Experience of the early phases of the UK pandemic

For almost all families, the experience of the pandemic during data collection had been one of the severe restrictions. A large proportion of both pre-adolescent and adolescent CYP did not access education as they usually would, even though it was UK policy that CYP with SEN (defined in the policy as someone with an Education Health and Care Plan) should have access to their usual educational placement (Skipp et al., 2021). We found no significant association between school attendance and CYP or parental mental health but given that some autistic CYP find school very stressful (Goodall, 2018), it is possible that school closures may have had a positive impact on mental health for some and a negative impact for others, cancelling each other out. This is in line with longitudinal research from Australia that showed school lockdowns were not associated with changes in mental health or feelings of loneliness for CYP with neurodevelopmental conditions (Houghton et al., 2022). However, lower engagement and enjoyment in education (remote or in-person) was associated with more CYP mental health symptoms (in both ASTAR QUEST) and parental mental health symptoms (in QUEST only). These relationships are consistent with the SLaM CAMHS study which focused on CYP with a variety of mental health problems (in preparation). While we do not know whether this is a pandemic-related or more general phenomenon, or indeed the direction of the relationship, it suggests that in more general terms there could be additional benefits (i.e. improved CYP and parental mental health) to any efforts that aim to improve autistic CYP’s engagement and enjoyment in education (or vice versa). In addition, many CYP were rarely getting outside and this was associated with more mental health symptoms among the adolescent sample and their parents. While not getting outside was viewed as an impact of the pandemic, again this finding may not be a pandemic-specific phenomenon and supports the more general recommendation that encouraging CYP to engage in physical activity (Ahn & Fedewa, 2011) and leave the house could be beneficial to both theirs and their parents’ mental health. In addition, a significant proportion of parents were furloughed from work, which may have had both positive (due to more time at home) and negative (loss of earnings, uncertainty about the future) impacts on mental health. In contrast to the clear impacts on everyday life, exposure to COVID-19 itself within both cohorts was minimal even though cases were high in the UK at the time and there was a very high level of concern (although case rates were lower than subsequent disease waves), with very few pre-adolescent or adolescent CYP, or their family members reporting suspected or positive COVID-19 cases.

### Factors associated with CYP mental health symptoms during the pandemic

For pre-adolescent autistic CYP, higher levels of pre-existing ADHD symptoms were associated with higher levels of behavioural/ADHD symptoms during the pandemic, perhaps simply highlighting the stability of symptoms within this domain over time (Carter Leno et al., 2022). This was also found in QUEST, but only at the bivariate level, where other factors were not controlled for. Of note, among both pre-adolescent and adolescent CYP, pre-existing emotional symptoms were not associated with pandemic-time emotional symptoms. One explanation could be that any pre-existing anxiety around the school was reduced during the pandemic because of school closures, but different factors that are not well identified in this study may be influencing this relationship. Another explanation could be this finding is related to the difficulties in measuring these types of problems in this population as autistic CYP often have difficulty communicating internal states (Kinnaird et al., 2019).

In contrast to our expectation, less observed restricted and repetitive behaviours were associated with greater pandemic-time behavioural/ADHD symptoms. This was consistent across both pre-adolescent and adolescent CYP. Given the inconsistencies between this finding and the relationship between social communication and interaction impairments and CYP’s mental health during the pandemic mentioned below, caution should be taken when interpreting this result. Different from the pattern of relationships between restricted and repetitive behaviours and CYP mental health, fewer parent-reported social communication and interaction impairments were associated with more emotional symptoms in the pre-adolescent children, but fewer emotional symptoms in the adolescent children during the pandemic. This could be due to the different ages of the two cohorts; the differential impact of the pandemic on emotional well-being for pre-adolescents versus adolescents; the role of school as a potential stressor for adolescents versus pre-adolescents; the use of different versions of the measure tapping into social communication and interaction impairments which asked about different time periods for the two cohorts (lifetime impairment for ASTAR versus current impairment for QUEST). Furthermore, the association between social communication impairment scores and CYP emotional symptoms in the pre-adolescent sample was non-significant at the bivariate level. Future research should be undertaken to replicate these effects.

In both cohorts, few familial or environmental stressors were associated with pandemic CYP mental health symptoms, except for pre-existing parental distress being associated with pandemic-time behavioural/ADHD symptoms among adolescents. This suggests that pre-existing mental health symptoms in both the child and parent are more pertinent to CYP mental health symptoms experienced during the pandemic than other environmental factors. This finding is consistent with pre-pandemic literature (Yorke et al., 2018).

### Factors associated with parental mental health symptoms during the pandemic

For parents of autistic CYP in our samples, more pre-existing parental distress was associated with increased pandemic-time mental health symptoms in parents of both pre-adolescent and adolescent children indicating the stability of these difficulties and vulnerability to pandemic impacts. More pandemic-time parental mental health symptoms were associated with increased financial concerns during the pandemic in parents of autistic adolescents; this relationship was replicated in the pre-adolescent cohort but only at the bivariate level when other factors weren’t controlled for. This finding is in line with the previous literature that demonstrated the impact of the pandemic on parents (Lengua et al., 2022; Yılmaz et al., 2021). Such impacts are expected given the substantial pressure social restrictions have placed on parents to deliver home-schooling and balance work demands, along with additional physical health, mental health and financial concerns. In both cohorts, few characteristics of the CYP were associated with pandemic parental mental health symptoms.

### Clinical implications

The combined findings from the two cohorts indicate that for autistic CYP and their parents, those more vulnerable to the impacts of the COVID-19 pandemic restrictions may have had heightened mental health symptoms pre-pandemic. This highlights the need for services to consider autistic CYP and their families’ pre-existing mental health symptoms when, for example, prioritising access (e.g. in the instance of another public health crisis, or if resources are limited for other reasons). Two types of continuity in mental health symptoms have been suggested, heterotypic and homotypic continuity (Angold et al., 1999). The findings from the current study suggest that in this particular sample, homotypic continuity was seen for behavioural/ADHD symptoms with earlier symptoms predicting later symptoms. This fits with other literature, demonstrating homotypic continuity of mental health symptoms in autistic CYP (Carter Leno et al., 2022) although with an overlapping sample to the current study (QUEST) using data collected prior to the pandemic. This suggests that the symptoms measured may partly represent a stable distint trait. However, the findings also suggest some heterotypic continuity from neurodevelopment traits to later mental health symptoms. This study also highlights the link between mental health and autistic CYP engagement with/enjoyment in education. Going forward, it will be important for schools to ensure that structures are in place to allow autistic CYP to engage in meaningful and enjoyable activities, whether these are delivered remotely or in-person. Furthermore, clinicians wishing to build on existing mental health interventions for autistic CYP may wish to incorporate opportunities for participation in activities (including physical exercise) outside of the home, for example, drawing on the growing evidence base for adaptive cognitive-behavioural therapies (Weston et al., 2016).

### Limitations

The survey used to assess the impact of the COVID-19 pandemic on CYP and their parents is not a standardised measure. This means that we cannot compare the levels of mental health symptoms among our samples with other samples or the population. In addition, measures of pre-existing mental health symptoms varied across the cohorts and over time. We therefore cannot make definite conclusions about whether mental health symptoms changed as a result of the pandemic and associated restrictions. In addition, the survey only provides a snapshot of experience as it asks about the 2 weeks prior to survey completion, and data were collected early in the pandemic. We do not know the prolonged impact of the restrictions or impacts when restrictions were re-imposed in the UK. However, there is growing recognition that referrals to CAMHS and evidence of increased CYP distress became apparent after the period of this survey completion (Huang & Ougrin, 2021). Furthermore, we did not ask about children’s educational placements during the pandemic but relied on reports provided previously. We expected education placements to be relatively stable between the data collection points in our samples as generally some movement from mainstream to specialist settings occurs as part of the primary to secondary school transition, while it is rare to move from specialist to mainstream. It is possible however that there is an overestimation of children in mainstream settings, particularly in the ASTAR cohort.

### Strengths and conclusions

This study used two well-characterised pre-existing cohorts to examine the effects of the UK pandemic on autistic children and parental mental health. The inclusion of these two cohorts, one a community sample and the other a sample involved in a feasibility pilot randomised controlled trial with few eligibility criteria drawn from similar geographical areas enabled us to understand what factors are consistently associated with mental health symptoms during the pandemic across pre-adolescent and adolescent CYP and their families. In summary, autistic CYP and their parents with greater pre-pandemic mental health symptoms are likely to be more at risk of having more pandemic-time mental health symptoms. This information should be used to guide care planning for autistic CYP and their families.

## Supplemental Material

sj-pdf-1-aut-10.1177_13623613231153694 – Supplemental material for Factors associated with mental health symptoms among UK autistic children and young people and their parents during the COVID-19 pandemic Supplemental material, sj-pdf-1-aut-10.1177_13623613231153694 for Factors associated with mental health symptoms among UK autistic children and young people and their parents during the COVID-19 pandemic by Melanie Palmer, Susie Chandler, Virginia Carter Leno, Farah Mgaieth, Isabel Yorke, Matthew Hollocks, Andrew Pickles, Vicky Slonims, Stephen Scott, Tony Charman and Emily Simonoff in Autism
