# Supplementary material for: Factors associated with mental health symptoms among UK autistic children and young people and their parents during the COVID-19 pandemic
Source: Autism. 2023 Feb 27;27(7):2098–111. doi: 10.1177/13623613231153694 (PMC9974376; doi:10.1177/13623613231153694)
Supplement: sj-pdf-1-aut-10.1177_13623613231153694 – Supplemental material for Factors associated with mental health symptoms among UK autistic children and young people and their parents during the COVID-19 pandemic [file sj-pdf-1-aut-10.1177_13623613231153694.pdf]

Title: Factors associated with mental health symptoms among UK autistic children and young people and their parents during the COVID-19 pandemic.

ASTAR Cohort

A total of 85 parents of young autistic children who consented to participate in a feasibility study or pilot randomised controlled trial (RCT) of novel group-based parent-mediations interventions (ISRCTN91411078) initially formed the sample. The Autism Spectrum Treatment and Resilience (ASTAR) pilot RCT (see Charman et al., 2021) was undertaken as part of the Improving Autism Mental Health (IAMHealth). Children with an existing clinical diagnosis of an autism spectrum disorder (ASD) were referred into the pilot RCT via local autism diagnostic teams, education professionals and support groups at participating services. Potential participants could also self-refer. To be eligible to take part, the children were required to have a clinical diagnosis of an autism spectrum disorder (including those classified by ICD-10 criteria: childhood autism, Asperger's syndrome, pervasive developmental disorder or atypical autism) and be between 4 years 0 months and 8 years 11 months. Given the high prevalence of co-occurring mental health problems in autistic children, there was no specific cut-off for inclusion in ASTAR based on the levels of emotional or behavioural symptoms the children displayed. Families were excluded if: they did not have sufficient spoken English to be able to take part in a group intervention; the child or parent had a severe hearing or visual impairment; the child had seizures more than once a week; there were active safeguarding concerns; or the parent had a current severe psychiatric disorder (see https://www.isrctn.com/ISRCTN91411078 for the trial record). Since intervention group composition and content of the interventions were adapted based on child verbal language (minimally verbal versus verbal expressive language), recruitment was

stratified by verbal ability. This meant that approximately 50% of the sample were minimally verbal and the other 50% were verbal.

Of the 85 parents who consented to take part in ASTAR, 83 participated in either the feasibility phase of the study (*n*=21 parent-child dyads) or the pilot RCT phase (*n*=62 parent-child dyads). In both phases of the ASTAR study, a range of clinical measures were completed by parents, children and teachers at baseline. Measures were completed again immediately following the group-based intervention. The present study is based on 67 of the original ASTAR cohort who completed the COVID survey during the pandemic (when aged 6:0–11:10 years).

QUEST Cohort

A total of 277 participants with an autism spectrum disorder initially formed the QUEST study, a longitudinal community sample recruited at age 4–8 years (wave 1) (Salazar et al., 2015) and followed up at ages 11–15 years (wave 2) and 12–17 years (wave 3), which in turn was also part of the wider IAMHealth programme. The target population for the study was all children born between 01/09/2000 and 31/08/2004, living in two London boroughs (one inner and one outer London), who had a clinical diagnosis of autism. 277 children were recruited into the study upon entry and split into an ‘intensively studied’ (hereafter intensives; *n*=101) and ‘extensively studied’ group (hereafter extensives; *n*=176). Participants in the intensive group were selected to over-represent females, as one of the main aims of the study included sex comparisons. This sampling structure was retained at wave 2 (*n*=211 for the total sample) and wave 3 (*n*=214 for the total sample). Although all participants had a clinical diagnosis of autism, the intensive group had their diagnosis confirmed at wave 2 with the Autism Diagnostic Observation Schedule–2 (ADOS–2) (Lord et al., 2012), and a subset also with the Autism Diagnostic Interview-Revised (ADI-R) (Rutter et al., 2003). Both the

recommended ASD cut-off (Rutter et al., 2003) and the recommended ASD cut-off (Risi et al., 2006) were applied to the ADI-R data. All participants were above threshold on either or both instruments. The present study is based on 112 (40%) of the original QUEST cohort who completed the COVID survey during the pandemic (when aged 15–20 years).

Imputation of ABAS GAC for QUEST participants completing only the Communication domain

QUEST intensives completed the full ABAS–2 (Harrison & Oakland, 2003), whereas extensives completed the Communication items only. Parent/carers of minimally verbal children completed the Communication items for 0-5 years, rather than items appropriate for their chronological age. For the extensives, scores for the other eight skill domains were calculated for the full sample using multiple imputation (100 imputations), with ABAS Communication scaled score, chronological age, school type along with the stratification variables used to select intensive cases at the start of the study (IQ, sex and SCQ score) included in the model. Imputations were only calculated where all variables included in the model were present. Imputed domain scores, based on the aggregated test statistic across the imputed datasets, were then used to generate general adaptive composite (GAC) scores for those who did not complete the full ABAS. This yielded ABAS GAC scores for 179 participants, of which 72 were based on observed domain scores and 107 were based on imputed domain scores. For the 72 with fully observed data, ABAS GAC and Communication scaled scores were highly correlated, $r(72)=.84$ ($p<.001$); and the prediction of ABAS GAC from the variables included in the imputation models was strong ($R^2=.74$, $F(7, 63)=26.23$, $p<.001$).

Attrition

There were no significant differences between ASTAR participants and non-participants with regard to the child's current age, verbal ability group (verbal/minimally verbal), adaptive functioning, level of autism characteristics (SCQ total score), or child education provision (mainstream/specialist as measured at initial assessment); nor regarding parental employment, parental education and household income at initial assessment. There were no significant differences between QUEST participants and non-participants with regard to young person's age during the initial lockdown, wave 1 IQ, wave 2 adaptive functioning, or wave 2 autism characteristics level (SCQ total score); or regarding parental education. However, parental unemployment at wave 2 was higher among QUEST non-participants (35%) when compared to those who participated (21%), $\chi^2(1, N=168) = 4.2, p=.040$).

Factor analysis

An exploratory factor analysis (EFA) using a minimum eigenvalue of 1 was conducted to explore the factor loadings of child mental health symptom items. For ASTAR, a two factor solution for the child mental health symptom items emerged which accounted for 94% of the variance in scores (see Supplementary Table 1 for factor loadings). For QUEST, the EFA resulted in a two-factor solution, accounting for 90% of the variance. Two factors were extracted which pertained to an Emotional Symptoms factor and a Behavioural and ADHD Symptoms factor. Items about infection-related worries were not used in the current analysis for either cohort.

For the parental mental health symptom items, a similar EFA was conducted. For this EFA, we specified the use of two factors (to determine whether a similar pattern to the child mental health factors emerged), loadings of at least 0.2 and a promax oblique rotation. For

ASTAR, this resulted in Emotional Symptoms and Irritable Behaviour factors that accounted for 103% of the cumulative variance in the scores. For QUEST, this resulted in a two factor model accounting for 100% of the variance (see Supplementary Table 2 for factor loadings).

Supplementary Table 1. Exploratory factor analysis for the COVID-19 child mental health symptoms items in the ASTAR and QUEST cohorts.

Item	ASTAR		QUEST	
	**Factor 1 - Emotional Symptoms**	**Factor 2 - Behavioural and ADHD Symptoms**	**Factor 1 – Emotional Symptoms**	**Factor 2 – Behavioural and ADHD Symptoms**
Child general worry	0.52		0.72	
Child sadness	0.77		0.63	
Child enjoyment of usual activities	0.51		0.23	
Child anxiety	0.75		0.74	
Child restlessness		0.23		0.45
Child tiredness	0.51		0.56	
Child concentration		0.20		0.41
Child irritability/anger		0.32		0.39
Child aggressiveness		0.44		0.53
Child loneliness	0.46		0.51	

Supplementary Table 2. Exploratory factor analysis for the COVID-19 parental mental health symptoms items in the ASTAR and QUEST cohorts.

**Item**	**ASTAR**		**QUEST**	
	**Factor 1 – Parent Emotional Symptoms**	**Factor 2 – Parent Irritable Behaviour**	**Factor 1 – Parent Emotional Symptoms**	**Factor 2 – Parent Irritable Behaviour**
Parent general worry	0.81		0.61	
Parent sadness	0.53		0.63	
Parent enjoyment of usual activities	0.48		0.48	
Parent anxiety	0.55		0.67	
Parent restlessness		0.56		0.55
Parent tiredness		0.81		0.58
Parent concentration		0.32		0.38
Parent irritability/anger		0.73		0.75

Supplementary Table 3. Measures used in the ASTAR and QUEST studies.

**Construct**		**ASTAR**		**QUEST**	
		**Measure used**	**Time-point**	**Measure used**	**Time-point**
CYP CHARACTERISTICS	Age	Age	During the pandemic	Age	During the pandemic
	Autism characteristics	ADOS–2 Social Affect Calibrated Severity Score (CSS)	Baseline	ADOS–2 Social Affect Calibrated Severity Score (CSS)	Wave 2
		ADOS–2 Restricted and Repetitive Behaviours Calibrated Severity Score (CSS)	Baseline	ADOS–2 Restricted and Repetitive Behaviours Calibrated Severity Score (CSS)	Wave 2
		SCQ-Lifetime Social Interaction and Communication score	Baseline	SCQ-Current Social Interaction and Communication score	Wave 2
		SCQ-Lifetime Restricted and Repetitive Behaviours score	Baseline	SCQ-Current Restricted and Repetitive Behaviours score	Wave 2
	Adaptive functioning	ABAS–3 General Adaptive Composite score	Baseline	ABAS–2 General Adaptive Composite score	Wave 2
CYP PRE-EXISTING MENTAL HEALTH SYMPTOMS	Emotional symptoms	PASR Anxiety score (Parent-report)	Post-intervention	SDQ Emotional symptoms score	Wave 3
	Disruptive behaviour symptoms	HSQ-ASD Non-Compliance Severity score (Parent-report)	Post-intervention	SDQ Conduct symptoms score	Wave 3

	ADHD symptoms	ABC Hyperactivity score (Parent-Teacher average)	Post-intervention	SDQ Hyperactivity/inattention symptoms score	Wave 3
PARENT PRE-EXISTING MENTAL HEALTH SYMPTOMS	Parental distress	APSI Parenting Stress score	Post-intervention	K-10 Parental Psychological Distress score	Wave 2
ENVIRONMENT	Financial concerns	Measured on a 5-point scale from 1 (Living comfortably) to 5 (Finding it very difficult)	During the pandemic	Measured on a 5-point scale from 1 (Living comfortably) to 5 (Finding it very difficult)	During the pandemic
	Home environment comfort level	Measured on a 5-point scale from 1 (Very comfortable) to 4 (Very problematic)	During the pandemic	Measured on a 5-point scale from 1 (Very comfortable) to 4 (Very problematic)	During the pandemic
	Personal garden access	Yes / No	During the pandemic	Yes / No	During the pandemic
	Child attending education in person at least part-time	Yes / No	During the pandemic	Yes / No	During the pandemic
	Enjoyment in education	Measured on a 5-point scale from 1 (Almost never) to 5 (Almost always)	During the pandemic	Measured on a 5-point scale from 1 (Almost never) to 5 (Almost always)	During the pandemic
	Engagement in education	Measured on a 5-point scale from 1 (Almost never) to 5 (Almost always)	During the pandemic	Measured on a 5-point scale from 1 (Almost never) to 5 (Almost always)	During the pandemic

	Child getting outside home for approved activities	Measured on a 4-point scale from 1 (Less than once a week) to 4 (Several times a day)	During the pandemic	Measured on a 4-point scale from 1 (Less than once a week) to 4 (Several times a day)	During the pandemic

ABAS–2 Adaptive Behaviour Assessment System – 2nd edition; ABAS–3 Adaptive Behaviour Assessment System – 3rd edition; ABC=Aberrant Behaviour Checklist; ADOS–2=Autism Diagnostic Observation Schedule – 2nd edition; APSI=Autism Parenting Stress Index; HSQ-ASD=Home Situations Questionnaire-Autism Spectrum Disorders; K-10=Kessler Psychological Distress Scale; PASR= Preschool Anxiety Scale Revised; SCQ=Social Communication Questionnaire; SDQ=Strengths and Difficulties Questionnaire.

Supplementary Table 4. Bivariate associations between the pre-existing and environmental factors and child and parent mental health symptoms for the ASTAR and QUEST cohorts.

	**ASTAR**				**QUEST**			
	CYP Emotional Symptoms	CYP Behavioural/ ADHD Symptoms	Parent Emotional Symptoms	Parent Irritable Behaviour	CYP Emotional Symptoms	CYP Behavioural/ ADHD Symptoms	Parent Emotional Symptoms	Parent Irritable Behaviour
	Std Coeff.	*Std Coeff.*	*Std Coeff.*	*Std Coeff.*	*Std Coeff.*	*Std Coeff.*	*Std Coeff.*	*Std Coeff.*
**CYP Characteristics and Autistic Symptoms**								
Age	0.20	-0.01	-0.05	0.08	0.00	0.05	-0.08	-0.03
SCQ Social Communication & Interaction	-0.20	0.07	0.17	0.17	0.04	0.24*	0.22*	0.16
SCQ Social Restricted and Repetitive Behaviours	0.02	0.14	0.14	0.18	-0.04	0.17	0.14	0.11
CYP ADOS–2 Social Affect CSS (on *n*=44 for QUEST)	0.08	0.03	-0.18	-0.10	-0.33*	0.03	0.56***	0.56***
CYP ADOS–2 Restricted and Repetitive Behaviours CSS (on *n*=44 for QUEST)	-0.19	-0.23*	-0.01	-0.08	-0.32*	-0.17	0.51***	0.53***
ABAS GAC Adaptive Functioning	0.12	-0.19	-0.09	-0.10	0.10	-0.08	-0.09	-0.08

**CYP Pre-existing Mental Health Symptoms**								
Emotional Symptoms	0.16	-0.00	0.00	-0.05	0.16	0.22*	0.09	0.07
Disruptive Behaviour Symptoms	0.29**	0.29**	0.22	0.30**	0.14	0.24**	0.21*	0.13
ADHD Symptoms	0.20	0.35**	0.15	0.35**	-0.04	0.30**	0.12	0.18
**Parent Pre-existing Mental Health Symptoms**								
Parental Distress	0.19	0.20	0.51***	0.46***	-0.04	0.30**	0.41***	0.34***
**Environmental Factors**								
Current Financial Concerns	0.15	0.15	0.35**	0.32**	0.14	0.30**	0.45***	0.37***
Current Home Environment Comfort Level	0.06	0.05	0.29**	0.20	0.08	0.15	0.25**	0.14
Personal Garden Access	-0.04	-0.03	-0.06	0.08	0.09	-0.02	-0.09	0.01
Accessing In Person Education	0.18	0.22	0.10	0.09	0.00	0.12	0.16	0.11

[a]ADOS–2 scores only available for 44/112 in QUEST
*, **, *** for p<.05, 01, .001

Supplementary Table 5. Associates of CYP mental health symptoms during the UK COVID-19 pandemic based on the bivariate associations: multivariate multiple regression results.

	**ASTAR (*N*=67)**		**QUEST (*N*=112)**	
	CYP Emotional Symptoms	CYP Behavioural and ADHD Symptoms	CYP Emotional Symptoms	CYP Behavioural and ADHD Symptoms
	Std Coeff, (p)	*Std Coeff, (p)*	*Std Coeff, (p)*	*Std Coeff, (p)*
CYP ADOS–2 Social Affect CSS	0.02, (.887)	0.02, (.886)	-0.31, (.074)	0.21, (.247)
CYP ADOS–2 Restricted and Repetitive Behaviours CSS	-0.12, (.328)	**-0.35, (.003)**	-0.34, (.126)	**-0.49, (.026)**
CYP ABAS GAC Adaptive Functioning	0.02, (.899)	**-0.32, (.020)**	-0.05, (.786)	-0.10, (.574)
CYP SCQ Social Communication & Interaction	**-0.33, (.011)**	-0.13, (.330)	**0.34, (.015)**	0.17, (.234)
Pre-existing CYP Emotional Symptoms	0.03, (.806)	0.00, (.974)	0.14, (.212)	0.16, (.166)
Pre-existing CYP Disruptive Behaviour Symptoms	0.30, (.069)	0.11, (.497)	0.09, (.478)	-0.06, (.654)
Pre-existing CYP ADHD Symptoms	0.08, (.552)	**0.29, (.027)**	**-0.33, (.013)**	0.14. (.282)
Pre-existing Parental Distress	0.04, (.794)	-0.12, (.415)	0.02, (.868)	**0.38, (.007)**
Current Financial Concerns	0.07, (.568)	0.11, (.335)	0.03, (.774)	0.16, (.177)

Note. Significant associations are bolded. Results in green indicate a significant association for both cohorts in the same direction; results in amber indicate a significant association in either cohort with associations going in the same direction in both cohorts; results in red indication a significant association in either cohort with associations going in the opposite direction across the cohorts.

Supplementary Table 6. Associates of parental mental health symptoms during the UK COVID-19 pandemic based on the bivariate associations: multivariate multiple regression results.

	**ASTAR (*N*=67)**		**QUEST (*N* = 112)**	
	Parent Emotional Symptoms	Parent Irritable Behaviour	Parent Emotional Symptoms	Parent Irritable Behaviour
	Std Coeff, (p)	*Std Coeff, (p)*	*Std Coeff, (p)*	*Std Coeff, (p)*
CYP ADOS–2 Social Affect CSS	**-0.26, (.011)**	-0.17, (.117)	0.11, (.447)	0.01, (.961)
CYP ADOS–2 Restricted and Repetitive Behaviours CSS	0.10, (.341)	-0.03, (.766)	-0.04, (.198)	-0.10, (.694)
CYP ABAS GAC Adaptive Functioning	0.07, (.497)	0.02, (.840)	-0.04, (.820)	-0.04, (.833)
Pre-existing CYP Disruptive Behaviour Symptoms	-0.20, (.137)	-0.10, (.497)	0.04, (.685)	-0.08, (.488)
Pre-existing CYP ADHD Symptoms	-0.05, (.661)	0.21, (.083)	-0.01, (.915)	0.10, (.442)
Pre-existing Parental Distress	**0.68, (<.001)**	**0.42, (.003)**	**0.26, (.019)**	**0.25, (.044)**
Current Financial Concerns	0.17, (.100)	0.14, (.224)	**0.38, (<.001)**	**0.28, (.008)**
Current Home Environment Comfort Level	0.09, (.397)	0.06, (.590)	0.11, (.273)	0.05, (.664)

Note. Significant associations are bolded. Results in green indicate a significant association for both cohorts in the same direction; results in amber indicate a significant association in either cohort with associations going in the same direction in both cohorts; results in red indication a significant association in either cohort with associations going in the opposite direction across the cohorts.

References

Charman, T., Palmer, M., Stringer, D., Hallett, V., Mueller, J., Romeo, R., Tarver, J., Perez, J. P., Breese, L., Hollett, M., Cawthorne, T., Boadu, J., Salazar, F., O'Leary, M., Beresford, B., Knapp, M., Slonims, V., Pickles, A., Scott, S., & Simonoff, E. (2021). A novel group parenting intervention for emotional and behavioral difficulties in young autistic children: autism spectrum treatment and resilience (ASTAR): A randomized controlled trial. *Journal of the American Academy of Child and Adolescent Psychiatry, 60.* 1404-1418.

Harrison, P., & Oakland, T. (2003). *Adaptive Behavior Assessment System (ABAS-2) - Second Edition*. The Psychological Corporation.

Lord, C., Rutter, M., DiLavore, P. C., Risi, S., Gotham, K., & Bishop, S. (2012). *Autism Diagnostic Observation Schedule* (2nd ed.). Western Psychological Services.

Risi, S., Lord, C., Gotham, K., Corsello, C., Chrysler, C., Szatmari, P., Cook, E. H.,Jr, Leventhal, B. L., & Pickles, A. (2006). Combining information from multiple sources in the diagnosis of autism spectrum disorders. *Journal of the American Academy of Child and Adolescent Psychiatry, 45*(9), 1094-1103. 10.1097/01.chi.0000227880.42780.0e

Rutter, M., Le Couteur, A., & Lord, C. (2003). *ADI-R: Autism Diagnostic Interview-Revised (ADI-R)*. Western Psychological Services.

Salazar, F., Baird, G., Chandler, S., Tseng, E., O'Sullivan, T., Howlin, P., Pickles, A., & Simonoff, E. (2015). Co-occurring psychiatric disorders in preschool and elementary school-aged children with autism spectrum disorder. *Journal of Autism and Developmental Disorders, 40*(9), 1080-1093. 10.1007/s10803-015-2361-5
